# Clinical value of video oculomotor evaluation in the differential diagnosis of multiple system atrophy and Parkinson's disease

**DOI:** 10.1002/brb3.3510

**Published:** 2024-05-07

**Authors:** Dongxiao Zhou, Qian Ma, Haiwei Huang, Xue Xu

**Affiliations:** ^1^ Department of Neurology, The First Affiliated Hospital, Sun Yat‐sen University, Guangdong Provincial Key Laboratory of Diagnosis and Treatment of Major Neurological Diseases National Key Clinical Department and Key Discipline of Neurology Guangzhou China; ^2^ Department of Neurology, The Fifth Affiliated Hospital Sun Yat‐sen University Zhuhai China

**Keywords:** multiple system atrophy, Parkinson's disease, video oculomotor evaluation

## Abstract

**Background:**

Multiple system atrophy (MSA) is a neurodegenerative disease that progresses rapidly and has a poor prognosis. This study aimed to assess the value of video oculomotor evaluation (VOE) in the differential diagnosis of MSA and Parkinson's disease (PD).

**Methods:**

In total, 28 patients with MSA, 31 patients with PD, and 30 age‐ and sex‐matched healthy controls (HC) were screened and included in this study. The evaluation consisted of a gaze‐holding test, smooth pursuit eye movement (SPEM), random saccade, and optokinetic nystagmus (OKN).

**Results:**

The MSA and PD groups had more abnormalities and decreased SPEM gain than the HC group (64.29%, 35.48%, 10%, *p *< .001). The SPEM gain in the MSA group was significantly lower than that in the PD group at specific frequencies. Patients with MSA and PD showed prolonged latencies in all saccade directions compared with those with HC. However, the two diseases had no significant differences in the saccade parameters. The OKN gain gradually decreased from the HC to the PD and the MSA groups (*p *< .05). Compared with the PD group, the gain in the MSA group was further decreased in the OKN test at 30°/s (Left, *p *= .010; Right *p *= .016). Receiver operating characteristic curves showed that the combination of oculomotor parameters with age and course of disease could aid in the differential diagnosis of patients with MSA and PD, with a sensitivity of 89.29% and a specificity of 70.97%.

**Conclusions:**

The combination of oculomotor parameters and clinical data may aid in the differential diagnosis of MSA and PD. Furthermore, VOE is vital in the identification of neurodegenerative diseases.

## Graphical abstract

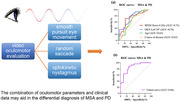

## 1 INTRODUCTION

Multiple system atrophy (MSA) is a sporadic adult‐onset neurodegenerative disease that manifests as a combination of Parkinson‐like symptoms, cerebellar ataxia, pyramidal tract, and autonomic dysfunction (Ozawa et al., 2004). There are differences between the pathophysiology and progression of MSA and Parkinson's disease (PD). However, symptoms overlap between the two conditions in the early stages, making clinical diagnosis difficult (Zhou et al., 2021). Traditional anti‐PD medications have limited effectiveness against MSA. Furthermore, developing novel neuroprotective treatments makes the early identification of MSA increasingly vital. However, there is a lack of available biomarkers for diagnosing MSA, particularly in its early stages.

Eye movement is a precise and meticulous activity of the human body, and video nystagmography (VNG) can objectively measure eye movements with the advantages of being precise, reproducible, and noninvasive. A growing number of studies have revealed that eye movement abnormalities recorded using VNG may be a potential biological indicator of Parkinsonism (Pinkhardt et al., 2009). PD and MSA are synucleinopathies, but their various oculomotor pathways result in different oculomotor manifestations owing to their distinct locations, with damage to the substantia nigra, corpus striatum, nuclei olivaris, nuclei pontis, and cerebellum (Armstrong, 2014; Gorges et al., 2018). Patients with PD have prolonged saccade latency, decreased saccade accuracy, and reduced smooth pursuit gain, which may involve a dysfunction of the frontal‐basal ganglia‐superior colliculus circuits associated with the course of disease and the severity of motor symptoms (Frei, 2021; Jung & Kim, 2019). Cerebellar‐related nystagmus, including positional nystagmus, gaze‐evoked nystagmus, and square‐wave jerks, strongly indicate the presence of MSA (Anderson et al., 2008). Such abnormalities can be identified during physical examinations, even during the early stages of the disease. Current studies are limited in assessing the role of oculomotor examination in differentiating between MSA and PD. This study aimed to explore the potential clinical value of oculomotor evaluation in the early differential diagnosis of MSA and PD.

## 2 METHODS

### 2.1 Participants

Notably, all participants were enrolled in the neurology ward of the First Affiliated Hospital of Sun Yat‐sen University between August 2018 and February 2023. The sample comprised (1) patients with MSA (*n* = 28), (2) patients with idiopathic PD (*n* = 31), and (3) healthy controls (HC) (*n* = 30). Three researchers made the diagnoses based on the criteria developed by the Movement Disorders Society and a consensus statement regarding MSA diagnosis (Gilman et al., 2008; Postuma et al., 2015). Patients with PD were assessed using the Unified PD Rating Scale (UPDRS) and Hoehn and Yahr staging. The scale scores and the subsequent oculomotor assessment of the patients with PD were performed in the OFF state. HC received no medication for movement disorder‐related diseases or other neurological disorders. All participants were free of substance abuse, vestibular system disorders, severe vision impairments, or any psychiatric disorders following the criteria stated in the Diagnostic and Statistical Manual of Mental Disorder‐V. Clinical information was obtained from each participant, including age, sex, course of disease, medication usage, and Mini‐Mental State Examination (MMSE) scores. The study was approved by the ethics committee of the hospital, and all participants provided a signed informed consent before participating in the study.

### 2.2 Video oculomotor evaluation

The eye movements of the enrolled patients were recorded using a 525 VNG (Interacoustics), a vestibular testing system including an eye‐tracking head‐mounted device and a series of test protocols. Participants stopped using drugs and alcohol that clearly or potentially affected vestibular function 48 h before the examination, and all tasks were performed in a dark room. During the examination, the participant was seated 1 m away from the screen, the head was fixed to minimize motion artefacts, and eye movements were recorded using a miniature infrared camera attached to the eye mask. Each participant's eye movements were calibrated using a red glowing dot on the screen as a visual target, and the participants were given verbal instructions to complete the task before starting. Notably, all participants had binocular movement conjugation, with no significant difference between the values of the left and right eyes, and the mean of the bilateral eye movement data was recorded. In addition, the quality of the test was ensured by examining all images and ruling out abnormalities due to lack of cognition or poor coordination.

#### 2.2.1 Spontaneous nystagmus and gaze‐holding test

The participants were asked to look straight ahead or gaze at the target in four positions (upward, downwards, left, and right; at 20° each), and eye movements were recorded over 20 s in each position. Spontaneous nystagmus, saccade intrusion, and gaze‐evoked nystagmus were also recorded.

#### 2.2.2 Smooth pursuit eye movement (SPEM)

Participants were asked to look at a horizontally or vertically moving target that made sinusoidal movements at a frequency of 0.2, 0.3, 0.4, and 0.5 Hz and an amplitude of ±15° (±10° in the vertical direction). The gain (the ratio of the peak velocity of eye movements to that of the target) and the left–right/up–down asymmetry ratio were recorded. Smooth sinusoidal curves that matched the visual target curve were considered normal (types I and II). Conversely, curves that were unsmooth, stepped, or completely disordered were considered abnormal (types III and IV). The classification of SPEM curves was semiquantitative.

#### 2.2.3 Random saccade

The visual target was randomly moved 30 times within a range of ±20° in the horizontal direction (±15° in the vertical direction) at fixed intervals (1−2 s). The participant's task was to move their gaze to the new target as quickly and accurately as possible. Peak velocity, latency, and accuracy of eye movements were recorded.

#### 2.2.4 Optokinetic nystagmus

Optokinetic nystagmus (OKN) refers to the movement of the eye in response to a full‐field visual stimulus, such as a series of black and white stripes moving horizontally across the participant's visual field at 30°/s and 40°/s for 10−15 s, with the gain and left–right asymmetry ratio recorded.

### 2.3 Statistical analysis

Data were analyzed using IBM SPSS Statistics 22. Categorical variables were analyzed using the chi‐square test or Fisher's exact tests, and the results were expressed as frequencies and percentages. One‐way analysis of variance was used for more than two groups of measurement data conforming to a normal distribution. Post hoc comparisons were adjusted for *p*‐values using Bonferroni correction, and comparisons between non‐normal measures were made using the Kruskal‐Wallis H test. Results were expressed as mean or median. A collinearity test of oculomotor parameters with statistical differences between MSA and PD was performed, and the independent correlation factors involved in the differential diagnosis were further analyzed using a binary logistic regression model.

## 3 RESULTS

### 3.1 Clinical data

Table 1 presents the clinical profiles of the 89 study participants and shows that the differences between the MSA and PD groups regarding age, sex, and course of disease were statistically insignificant. There was a significant difference in the MMSE scores among the three groups; however, the mean was above normal, and all participants performed well in all tests without intellectual or psychiatric abnormalities. Patients in the PD group were generally treated with levodopa at the time of the study but at a different dose than that administered to patients with MSA (*p *= .015).

**TABLE 1:** Demographic and clinical characteristics of participants.

	MSA (*n *= 28)	PD (*n *= 31)	HC (*n *= 30)	*p* Value
Age (years)	55.18 ± 8.26	60.35 ± 10.81^b^, ^d^	53.43 ± 9.91	.019^a^
Sex (M/F)	17/11	14/17^b^, ^d^	14/16	.428^a^
Course of disease (months)	16.5 (12, 33)	36 (12, 48)	–	.077^b^
Hoehn and Yahr stage	–	2 (2, 3)	–	–
UPDRS motor part	–	15 (8, 25)	–	–
MMSE	25.32 ± 3.56	27.12 ± 2.22^b^, ^c^	28.10 ± 1.75	.000^a^
LEDD (mg)	0 (0, 375)	375 (250, 375)	–	.015^b^
Spontaneous nystagmus, *n* (%)	1 (3.57)	1 (3.23)^b^, ^d^	3 (10.00)	.440^a^
Saccade intrusion, *n* (%)	10 (35.71)	9 (29.03)^b^, ^d^	2 (6.67)	.023^a^
Gaze‐evoked nystagmus, *n* (%)	4(14.29)	2 (6.45)^b^, ^d^	2(6.67)	.496^a^
SPEM III/IV, *n* (%)	18 (64.29)	11 (35.48)^b^, ^c^	3 (10.00)	.000^a^

Abbreviations: F, female; HC, healthy controls; LEDD, levodopa equivalent daily dose; M, male; MMSE, Mini‐Mental State Examination; MSA, multiple system atrophy; OKN, optokinetic nystagmus; PD, Parkinson's disease; SPEM, smooth pursuit eye movement.

^a^ MSA compared with PD and HC.

^b^ MSA compared with PD.

^c^ *p <* .05.

^d^ *p *> .05. mean ± SD, median (interquartile range).

### 3.2 Spontaneous nystagmus and gaze‐holding test

Spontaneous nystagmus and gaze‐evoked nystagmus frequencies were low in all groups, with no statistical differences (Table 1). Saccade intrusion was observed in all three groups and was significantly increased in the MSA and PD groups compared with the HC group (*p *= .023), but not between the case groups (10 [35.71%], 9 [29.03%]), respectively.

### 3.3 SPEM

**FIGURE 1:**
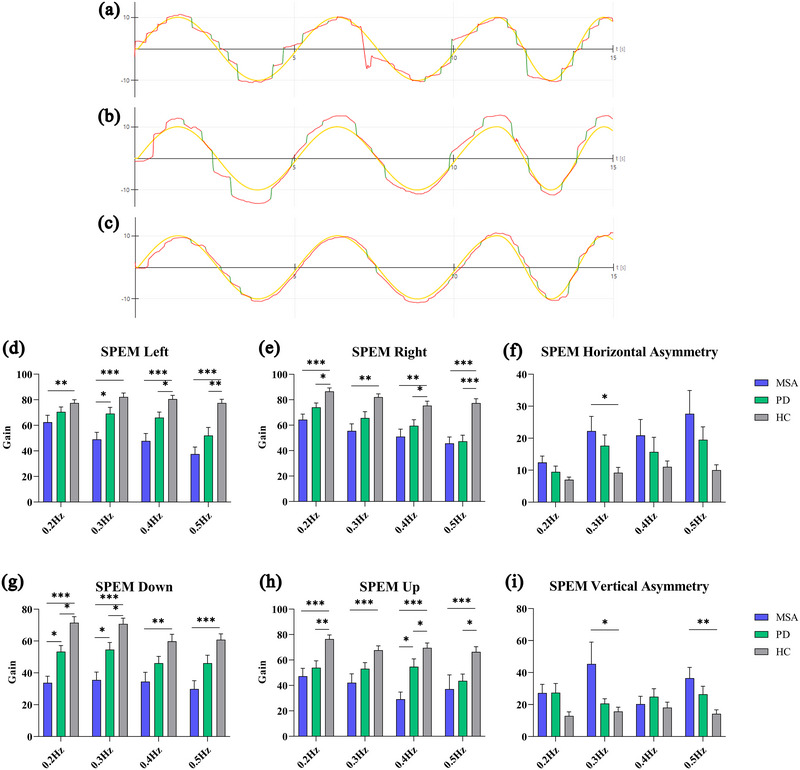
Illustrations of the patient's eye movement (red curve) during the vertical smooth pursuit test and comparison of the smooth pursuit eye movement (SPEM) test in each direction among groups. (a) Abnormal eye movement performance of a patient with multiple system atrophy (MSA). (b) Abnormal eye movement performance of a patient with Parkinson's disease (PD). (c) Normal performance of healthy control (HC). Green curve, saccades. * p < 0.05, ** p < 0.01, *** p < 0.001.

Patients in the MSA and PD groups generally had poor oculomotor tracking (Figure 1), resulting in a marked increase in the frequency of abnormal oculomotor trajectories (type III, type IV) (Table 1), especially in the MSA group (MSA, 64.29%; PD, 35.48%; *p *< .001). As shown in Figure 1, the SPEM gain of the MSA group decreased compared with that of the HC group at all frequencies in the horizontal or vertical direction, whereas the SPEM gain of the PD group was lower than that of the HC group only at frequencies in the horizontal (Left 0.4 and 0.5 Hz; Right 0.2, 0.4, and 0.5 Hz) and vertical directions (Down 0.2 and 0.3 Hz; Up 0.2, 0.4, and 0.5 Hz). Furthermore, the reduction in SPEM gain is more pronounced in the MSA group than in the PD group in the horizontal (Left 0.3 Hz, *p *= .032) and vertical directions (Down 0.2 Hz, *p *= .032; 0.3 Hz, *p *= .011; Up 0.4 Hz, *p *= .030). In addition, the asymmetry ratio results (Figure 1i) indicated that patients with MSA may have differences in the vertical direction of eye movement compared with HCs.

### 3.4 Random saccade

**FIGURE 2:**
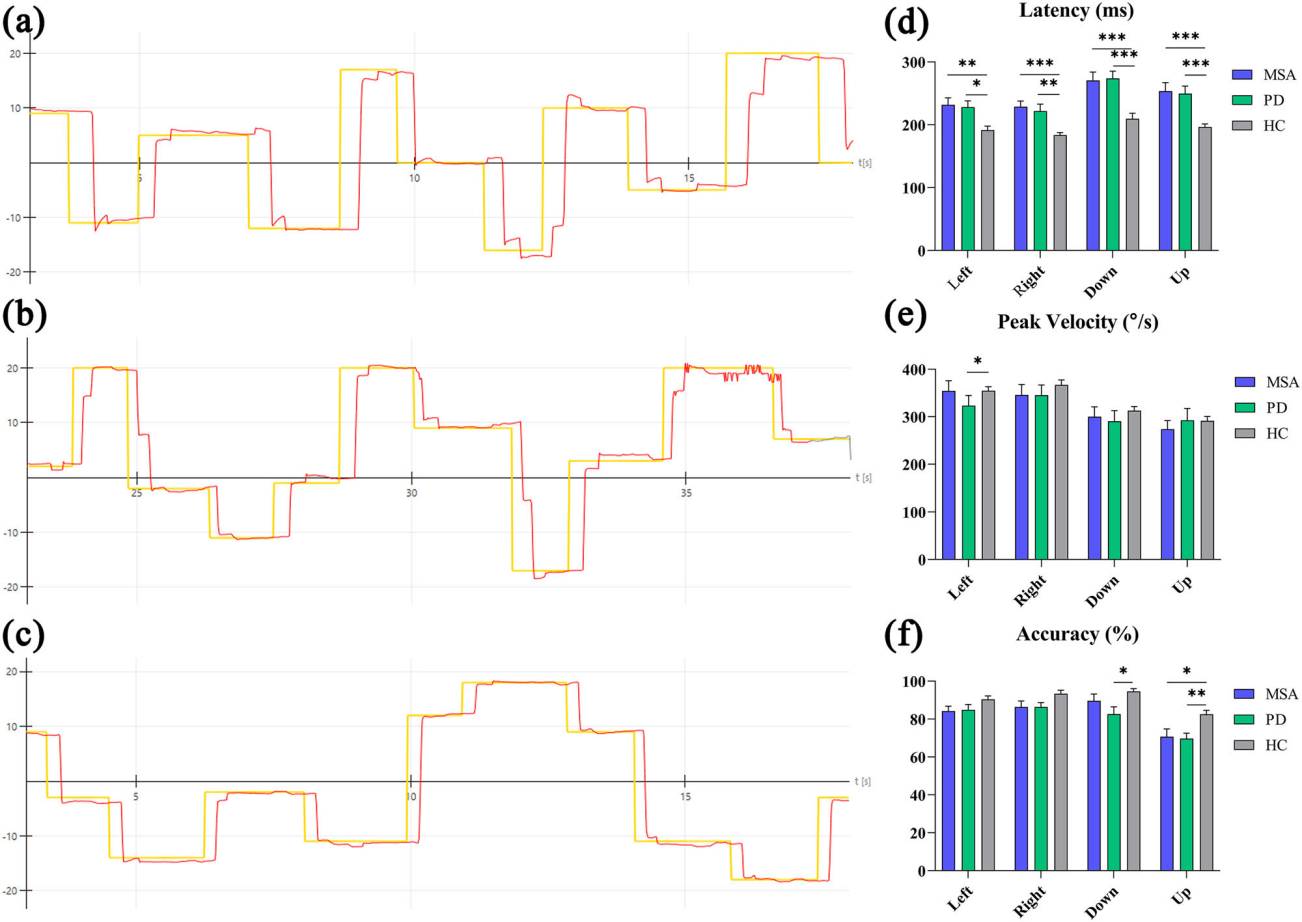
Illustrations of the patient's eye movement (red trajectory) during the random saccade test and comparison of the results of latency, peak velocity, and accuracy of the random saccade test in each group. (a) A patient with MSA's (multiple system atrophy) saccadic eye movement. (b) Example of saccade measurement in a patient with Parkinson's disease (PD). (c) An HC's (healthy control) result in random saccade test. * p < 0.05, ** p < 0.01, *** p < 0.001.

The MSA and PD groups showed prolonged latencies in all directions of the saccade compared with the HC group (*p *< .05, Figure 2d), with a significant decrease in leftward peak velocity and vertical accuracy in the PD group and a decrease in upward accuracy in the MSA group (*p *< .05). However, no significant differences were found between the MSA and PD groups for the saccade parameters (*p *> .05).

### 3.5 OKN

**FIGURE 3:**
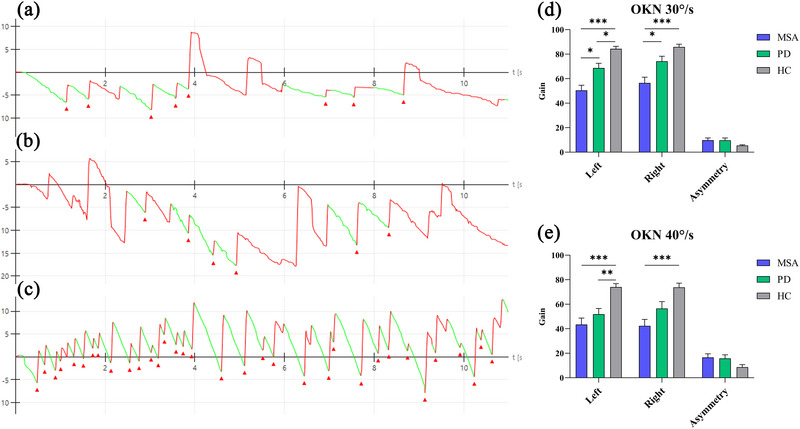
Recordings illustrating the patient's nystagmus during the optokinetic test and comparison of the optokinetic test in horizontal direction among the three groups. (a) A patient with MSA's (multiple system atrophy) nystagmus induced by the optokinetic test. (b) A patient with PD's (Parkinson's disease) nystagmus induced by the optokinetic test. (c) Reflex nystagmus produced by the eyeballs tracking a black‐and‐white frame moving continuously and rapidly in healthy control (HCs). OKN, optokinetic nystagmus. * p < 0.05, ** p < 0.01, *** p < 0.001.

In the horizontal OKN examination, only the HC group exhibited stable reflexive nystagmus (Figure 3c), and the gain gradually decreased in value from the HC to the PD and the MSA groups (Figure 3d). Further pairwise comparisons revealed that, compared to the PD group, the gain in the MSA group decreased in the OKN test at 30°/s (Left 30°/s, *p *= .010; Right 30°/s, *p *= .016). The asymmetry of the OKN test in the horizontal direction was not significantly different.

### 3.6 MSA‐C and MSA‐P

**FIGURE 4:**
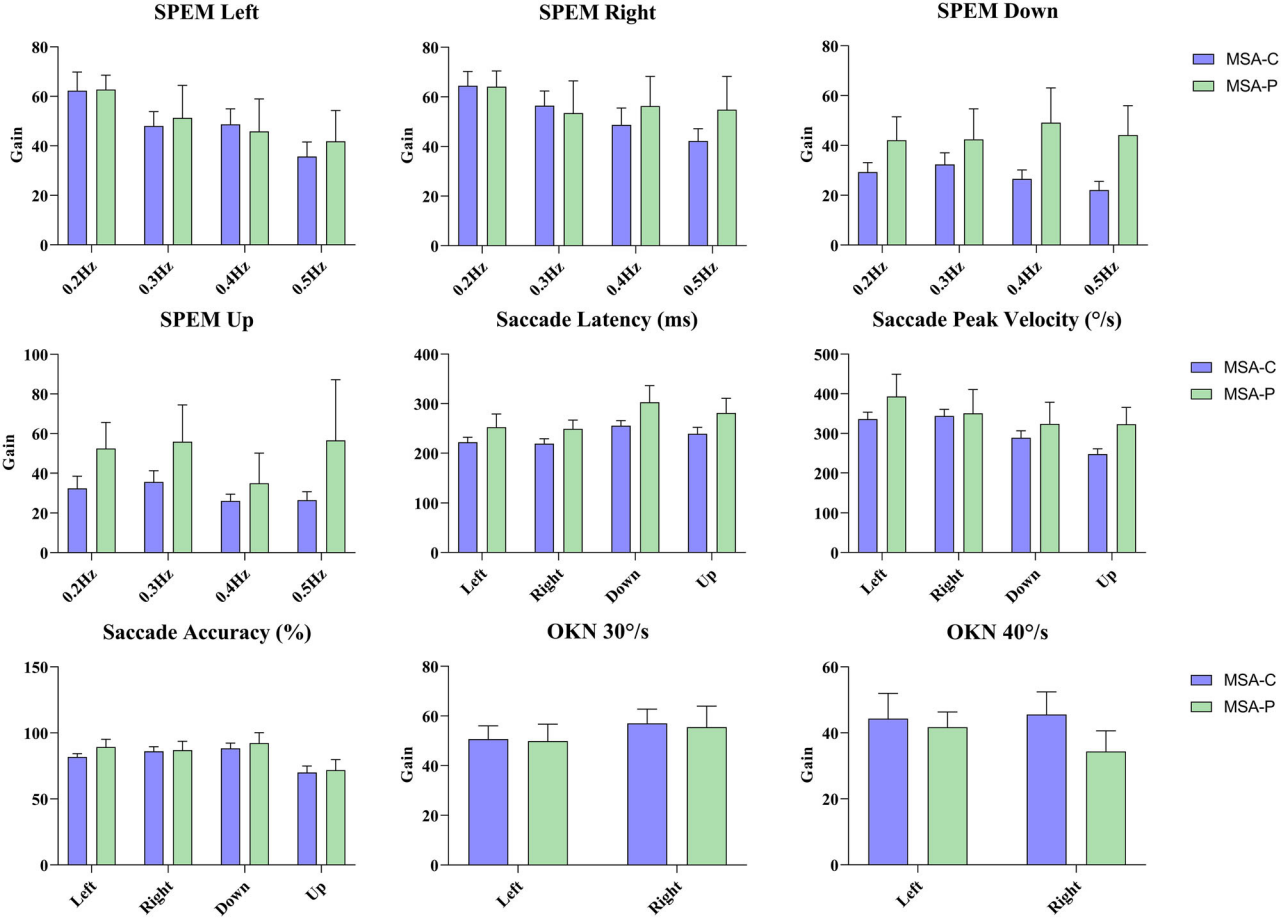
Comparison of the results of all parameters in smooth pursuit, random saccade, and optokinetic test in patients between groups multiple system atrophy of cerebellar type (MSA‐C) and multiple system atrophy of Parkinsonian type (MSA‐P). OKN, optokinetic nystagmus; SPEM, smooth pursuit eye movement.

The enrolled patients with MSA were further divided into MSA of cerebellar type (MSA‐C) (*n *= 19, 67.86%) and MSA of Parkinsonian type (MSA‐P) (*n *= 9, 32.14%) groups based on clinical manifestations. Further statistical analysis of the two MSA subtypes revealed no significant differences in oculomotor parameters (Figure 4).

### 3.7 Receiver operating characteristics

**FIGURE 5:**
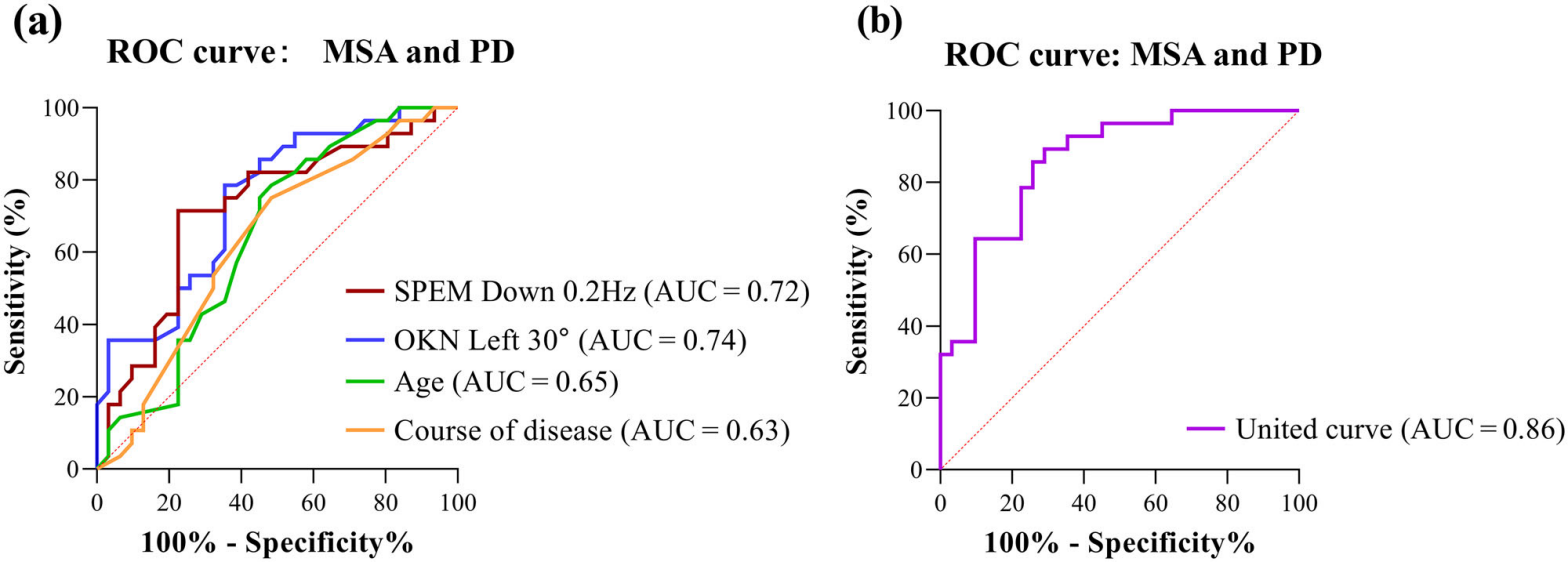
Receiver operating characteristic (ROC) curve associated with the differential diagnosis of multiple system atrophy (MSA) and Parkinson's disease (PD). (a) ROC of four parameters distinguish MSA from PD (smooth pursuit eye movement [SPEM] down 0.2 Hz, optokinetic nystagmus [OKN] left 30°/s, and age and course of disease). (b) ROC for the united prediction probability. AUC, area under the curve.

The six parameters (SPEM Left 0.3 Hz; SPEM Down 0.2 and 0.3 Hz; Up 0.4 Hz; OKN Left 30°/s; OKN Right 30°/s) that are statistically different between the MSA and PD groups in the oculomotor evaluation were tested for collinearity with clinical data such as age, sex, and course of disease, and no multiple multicollinearity was observed between multiple parameters (Variance Inflation factor, VIF < 10). Therefore, a binary logistic regression model was constructed, and the Hosmer–Lemeshow goodness‐of‐fit test showed *p *= .60 > .05. The SPEM Down 0.2 Hz, OKN Left 30°/s, age, and course of disease were independent correlation factors for diagnosing MSA and PD. The smaller the corresponding eye movement gain, the more likely MSA will be diagnosed. Further receiver operating characteristics (ROC) analysis of the above parameters showed that SPEM Down 0.2 Hz: area under the curve (AUC) = 0.72, *p *= .004; OKN Left 30°/s: AUC = 0.74, *p *= .001; Age: AUC = 0.65, *p *= .056; Course of disease: AUC = 0.63, *p *= .081. It can be seen that the two oculomotor parameters have good independent test efficacy. By fitting the above four indicators, ROC analysis suggested that it helped to identify MSA and PD with a sensitivity of 89.29%, a specificity of 70.97%, and an AUC of 0.86, 95% confidence interval (CI) (0.76, 0.95), *p *< .001 (Figure 5).

## 4 DISCUSSION

Currently, several clinicians have identified PD, MSA, and other movement disorders using video oculomotor evaluation (VOE) (Herwig et al., 2021; Marx et al., 2012; Wunderlich et al., 2021; Zhou et al., 2021); however, the research is generally limited to individual oculomotor parameters, such as SPEM or saccade, and the diagnosis is mainly qualitative (Gorges et al., 2016; Pinkhardt et al., 2009; Termsarasab et al., 2015). From the perspective of the characteristic manifestations of eye movements, we found that spontaneous nystagmus, saccade intrusion, and gaze‐evoked nystagmus were low and of limited clinical value. Patients with MSA have reported more specific nystagmus, such as positional nystagmus and square‐wave jerk (Pinkhardt & Kassubek, 2011), which was one of the focuses of our follow‐up studies. This study aimed to present a quantitative analysis of oculomotor parameters, such as SPEM, random saccade, and OKN, combined with clinical data, such as age and course of disease, to jointly differentiate the diagnosis of MSA and PD.

Previous studies have shown that neural circuits between structures such as the globus pallidus, caudate nucleus, thalamus, and basal ganglia are crucial in regulating and controlling SPEM (Cui et al., 2003; Tanaka, 2005; Yoshida & Tanaka, 2009). Notably, various oculomotor changes in patients with PD may be associated with abnormalities in the basal ganglia or nigrostriatal pathways. Furthermore, several studies have demonstrated reductions in SPEM gain and saccade abnormalities (Frei, 2021). The participants included in this study were previously diagnosed patients with PD who had been treated with dopamine. However, the effect of dopamine on oculomotor deficits is currently inconclusive, and it is hypothesized that oculomotor deficits in patients with PD are associated with the dysfunction of the non‐dopaminergic system (Pinkhardt et al., 2012). The cerebellum is also crucial in smooth pursuit, especially in the structures of the flocculus‐paraflocculus, ocular motor vermis (OMV), caudal fastigial nuclei (CFN), and ansiform lobule, involving the nodulus and uvula. The paraflocculus receives signal afferents from several structures to control smooth pursuit, with the OMV encoding speed and the CFN, nodulus, and ventral uvula being active in the downward SPEM (Beh et al., 2017). Our study also found a significant difference in SPEM at specific frequencies in patients with MSA compared with those with PD, and there may be a difference in the reduction of gain in the vertical direction, especially in the downward direction. Given the pathophysiological mechanisms underlying MSA, regardless of the complex or overlapping clinical features resulting from olivopontocerebellar or nigrostriatal degenerations, patients with MSA may have more severe oculomotor deficits than those with PD. The reduction in the SPEM gain was more pronounced in patients with Parkinson's syndrome than in those with PD (Vidailhet et al., 1994).

It has been shown that patients with PD differ from healthy participants in parameters such as latency and saccade accuracy (Zhang et al., 2021). The nerve fibers associated with eye movement project to the superior colliculus (SC) through the basal ganglia substantia nigra pars reticulata, which exerts a sustained inhibitory effect on the SC, and the abnormal saccades of PD may be associated with the weakened inhibition of SC by the apoptosis of dopaminergic neurons in the substantia nigra (Calabresi et al., 2014; Chevalier et al., 1981; Hikosaka et al., 2000). Our study also found that patients with PD had abnormalities in several indicators of saccade testing compared with HCs, whereas patients with MSA were similar to PD. The clinical manifestations of the two types of MSA are inconsistent; however, their pathophysiology has been changing and progressing (Terao et al., 2016). It has been suggested that the oculomotor changes in patients with MSA‐P and MSA‐C are similar, consistent with our findings, probably because all patients with MSA have coexisting nigrostriatal degeneration and cerebellar lesions (Pinkhardt et al., 2009).

OKN is a reflexive eye movement induced by a full‐field stimulus that stimulates the smooth pursuit system (fovea) and optokinetic system (peripheral retina) (Tarnutzer & Straumann, 2018). Whether PD affects OKN remains controversial. Current studies have found that impaired OKN may be associated with sensory perception, attention, and other factors, in addition to impairment of the optokinetic pathway in patient with PD; however, some studies have shown that patients with PD have intact OKN (Fujiwara et al., 2017). The optokinetic pathway involves the cerebral cortex, basal ganglia, cerebellum, and brainstem, making eye movement abnormalities more common in MSA. The incidence of OKN deficits was significantly higher in patients with MSA than in HCs, with reduced OKN gain (Zhou et al., 2022). The present study found that OKN gain in the horizontal direction was significantly reduced in patients with MSA compared with HCs and also differed from that in patients with PD. It is clear from the ROC that OKN may also have the potential to be used as an electrophysiological indicator to differentiate MSA from PD. However, whether there is a difference in left or right OKN still needs to be followed up by increasing the sample size and removing more confounding factors.

Oculomotor evaluation is now widely used to study neurodegenerative diseases (Gorges et al., 2014); however, due to the complexity of the vestibular pathways involved, a single oculomotor parameter can provide different results or interpretations in different experiments. It is difficult to represent all patients with PD and MSA with the small sample size in this study, but we combined SPEM, OKN, as well as age, course of disease, and the co‐analysis of other indicators through the ROC curve analysis, which showed that the multifactorial joint identification of the two diseases has a high sensitivity and specificity, to quantitatively construct a clinical model that is more convenient for clinical application. Furthermore, VOE has the advantages of being noninvasive, easy to perform, and inexpensive; therefore, we believe that VOE has high clinical application value in the differential diagnosis of MSA, PD, and other neurodegenerative diseases.

## AUTHOR CONTRIBUTIONS

All authors contributed to the conception and design of the study. Data collection, statistical analysis and manuscript drafting were performed by Dongxiao Zhou and Qian Ma. Xue Xu and Haiwei Huang were responsible for the critical revision of the manuscript.

## CONFLICT OF INTEREST STATEMENT

The authors declare no conflicts of interest.

### PEER REVIEW

The peer review history for this article is available at https://publons.com/publon/10.1002/brb3.3510.

## Data Availability

Data supporting the findings of this study are available from the corresponding author upon reasonable request.
